# Genomic Variations Between Fibrolamellar and Conventional Hepatocellular Carcinomas

**DOI:** 10.7759/cureus.50795

**Published:** 2023-12-19

**Authors:** Bengi Öztürk

**Affiliations:** 1 Gastroenterology Department, Hacettepe University Faculty of Medicine, Ankara, TUR

**Keywords:** structural variants, mutation, genomic variation, conventional hepatocellular carcinoma, fibrolamellar hepatocelullar carcinoma

## Abstract

Aim

The aim of this study is to define genomic variations between fibrolamellar hepatocellular carcinoma (FL-HCC) and conventional hepatocellular carcinoma (HCC)

Methods

This study used the American Association for Cancer Research (AACR) Project GENIE data as a foundational element. Specifically, information about both fibrolamellar and conventional hepatocellular carcinoma was retrieved from this database.

Results

A total of 719 patients diagnosed with HCC and 52 individuals presenting with FL-HCC underwent thorough analysis. Notably, distinct variances in gene alterations were observed between the two cohorts. Predominantly, the HCC group exhibited frequent occurrences of mutations within the *TP53 *and *CTNNB1 *genes. Conversely, DNAJB1 fusion was uniquely identified in FL-HCC cases.

Conclusion

This study significantly broadens our understanding of the genetic makeup associated with FL-HCC and HCC. It is particularly notable because it reveals clear disparities in gene modifications between FL-HCC and HCC. Further investigation is essential to unravel the functional consequences of these genetic variances. This exploration will aid in the development of targeted therapeutic approaches to enhance the prognosis of patients diagnosed with diverse subtypes of HCC.

## Introduction

Hepatocellular carcinoma (HCC) ranks as the sixth most prevalent cancer and stands as the third leading cause of death among all cancer types, according to GLOBOCAN data [1]. Fibrolamellar HCC (FL-HCC), one of the HCC subtypes, is a rare subtype but differs significantly from other HCC subtypes. FL-HCC is often seen in young people and does not have a background of chronic inflammation and fibrosis. The etiology of FL-HCC remains unknown. Elevated alpha-fetoprotein (AFP) is observed in HCC, but in FL-HCC, AFP is normal [2,3]. FL-HCC more often presents with large tumor size, lymph nodes, and distant metastases. The primary treatment is surgery [4-6]. The effectiveness of immunotherapy or tyrosine kinase inhibitors, which have been found to be effective in conventional HCC in metastatic cases, has not been demonstrated in randomized controlled studies in FL-HCC [7]. FL-HCC has conventionally been regarded as biologically less aggressive than conventional HCC, which is attributed to its favorable overall survival rates. However, this improved prognosis might predominantly stem from a younger patient cohort without underlying liver disease, facilitating a considerably larger proportion of patients eligible for potentially curative surgical resection [8,9]. The histological and immunohistochemical profile of FL-HCC also varies. Upon microscopic evaluation, the observed tumors manifest distinctive histological features characterized by the presence of enlarged cellular structures exhibiting eosinophilic cytoplasm, centrally positioned enlarged nuclei, and a discernible central lamellated scar encapsulating these sizable cellular entities [7]. Furthermore, the immunohistochemical profile unique to FL-HCC reveals marked positivity for specific biomarkers, notably the biliary marker CK7 and the histiocytic marker CD68 [10]. In addition to clinical and histological changes, they show differences at the genomic level. At the molecular level, FL-HCC is characterized by a distinct fusion between DNAJB1 and PRKACA located on chromosome 19. This fusion occurs because of a chromosomal deletion that intervenes between these two genes [11]. The identified fusion has demonstrated absolute specificity and is exclusively associated with FL-HCC within the spectrum of hepatic malignancies [12,13].

In this study, we showed the molecular differences between fibrolamellar HCC and conventional HCC.

## Materials and methods

The AACR Project GENIE, an initiative by the American Association for Cancer Research (AACR), is a comprehensive, multi-phase, and international effort aimed at advancing precision cancer medicine [10-12]. For this study, data were derived from AACR Project GENIE, specifically version 14.1, encompassing a public cohort of 160,965 patients [12,13]. Rigorous protocols were followed to obtain permissions ensuring patient privacy, sanctioned by the local Institutional Review Boards (IRBs) of participating institutions. The consortium operates under two key legal frameworks: a master participation agreement and a data use agreement. The former mandates data sharing among institutions adhering strictly to patient consent and specific IRB policies. Data-sharing methods encompass various approaches, including IRB-approved prospective patient consent, retrospective IRB waivers, and IRB approvals of GENIE-specific research proposals [12]. Anonymized data contributions were provided by participating institutions. The GENIE Project database integrates Clinical Laboratory Improvement Amendments (CLIA) and International Organization for Standardization (ISO)-certified genomic data sourced systematically from diverse international institutions. For in-depth information, detailed resources are available at cbioportal.org. The study's referenced data were extracted from the cBioPortal for cancer genomics (genie.cbioportal.org).

The genetic information encompasses mutations, variations in copy numbers, and structural irregularities. All genetic analyses were conducted utilizing modern next-generation sequencing methods. Data were gathered from version 14.1 of the GENIE project's cohort. The cBioPortal for cancer genomics (genie.cbioportal.org) was utilized to access this data. Patients diagnosed with HCC (hepatocellular carcinoma) and fibrolamellar carcinoma were identified based on the OncoTree cancer type classification. From these patients, we extracted fundamental characteristics such as gender, race, and the count of mutations in each individual, alongside their genetic data including mutations, variations in copy numbers, and structural irregularities, all of which were compiled into a database.

Statistical analysis

The dataset underwent descriptive analysis, presenting continuous variables through median and interquartile range (IQR), while categorical variables were expressed as percentages. Categorical variable comparisons involved Kruskal-Wallis or chi-square tests. Wilcoxon test was utilized for comparing paired groups. To ensure precision, the Benjamini-Hochberg method corrected p-values, calculating false discovery rate-adjusted q-values. Any q-value below 0.05 indicated statistical significance for associated p-values less than 0.05.

## Results

A total of 745 samples of 719 patients with HCC and 54 samples of 52 patients with FL-HCC were analyzed from the AACR Project GENIE data version 14.1 public cohort. The data centers are shown in Table 1.

**Table 1:** Data centers The data is conveyed in terms of numbers (N) and percentages (%).

Center	HCC	FL-HCC
Memorial Sloan Kettering Cancer Center	319 (44.4%)	38 (73.1%)
Dana-Farber Cancer Institute	87 (12.1%)	4 (7.7%)
Providence Health & Services Cancer Institute	66 (9.2%)	1 (1.9%)
Herbert Irving Comprehensive Cancer Center, Columbia University	51 (7.2%)	-
University of Texas MD Anderson Cancer Center	40 (5.6%)	-
University of California, San Francisco	38 (5.3%)	4 (7.7%)
Princess Margaret Cancer Centre, University Health Network	30 (4.2%)	2 (3.8%)
Vanderbilt-Ingram Cancer Center	26 (3.2%)	-
Institut Gustave Roussy	15 (2.1%)	1 (1.9%)
Johns Hopkins Sidney Kimmel Comprehensive Cancer Center	12 (1.7%)	-
Wake Forest Baptist Medical Center, Wake Forest University Health Sciences	11 (1.5%)	-
Duke Cancer Institute, Duke University Health System	10 (1.4%)	-
Yale Cancer Center, Yale University	7 (1.0%)	-
Swedish Cancer Institute	4 (0.6%)	-
Vall d’Hebron Institute of Oncology,	2 (0.3%)	2 (3.8%)
Netherlands Cancer Center	1 (0.1%)	-

**Figure 1:**
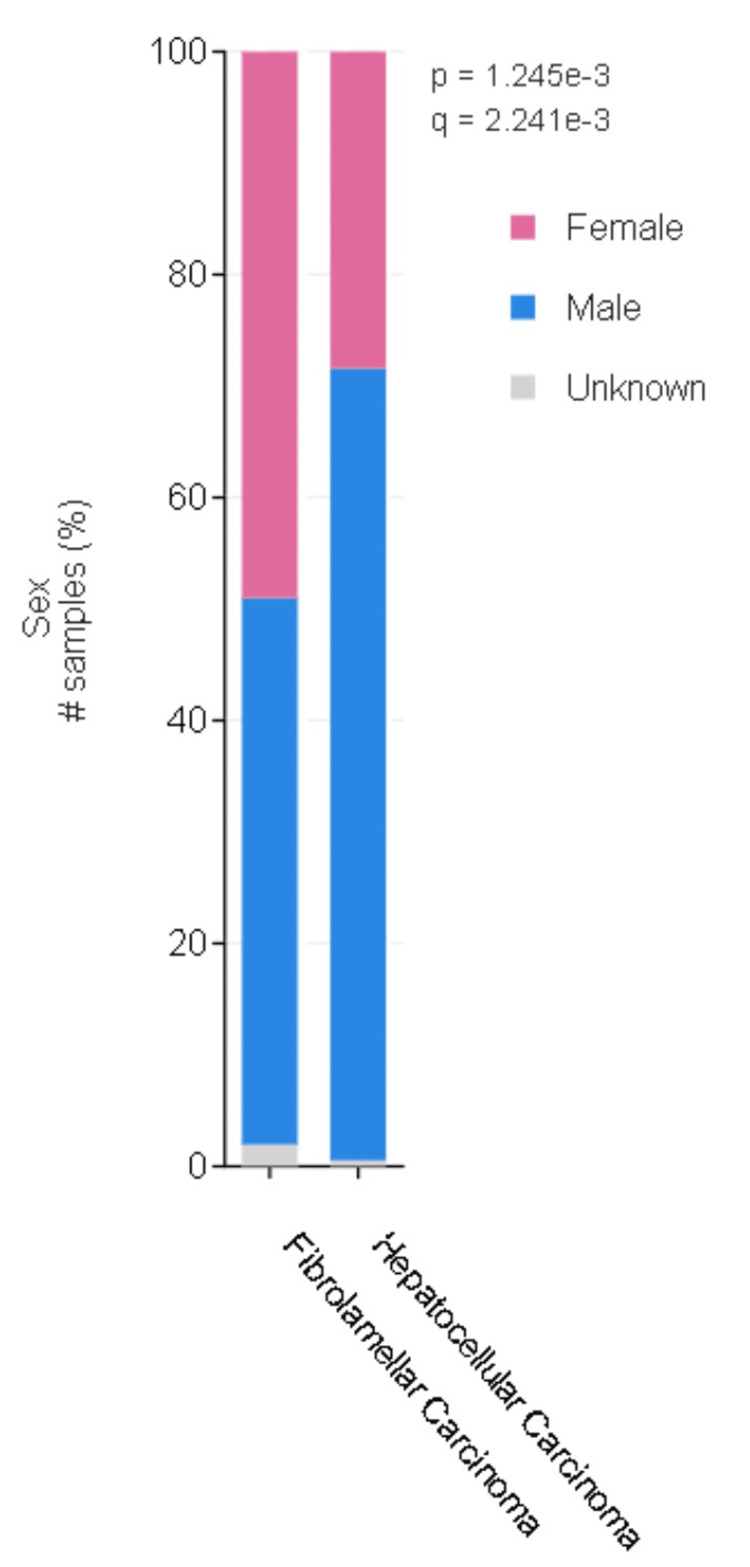
Gender differences in fibrolamellar hepatocellular carcinoma and hepatocellular carcinoma Blue bars represent male and pink bars represent female. The data has been represented as percentages (%). Difference between the groups was statistically significant (q<0,05).

In the HCC group, 512 patients were male (71.2%) and 204 patients were female (28.4%). 542 (72.8%) samples were from the primary tumor. The majority of patients were Caucasian (61.8%). In the FL-HCC group, 25 patients were male and 26 patients were female. 30 samples (55.6%) were from the primary tumor. The majority of patients were Caucasian (76.9%). The gender difference between FL-HCC and HCC was statistically significant (p<0.05). In the conventional HCC group, the majority of the patients were males, but in the FL-HCC group, the number of males and females was similar (Figure 1).

**Figure 2:**
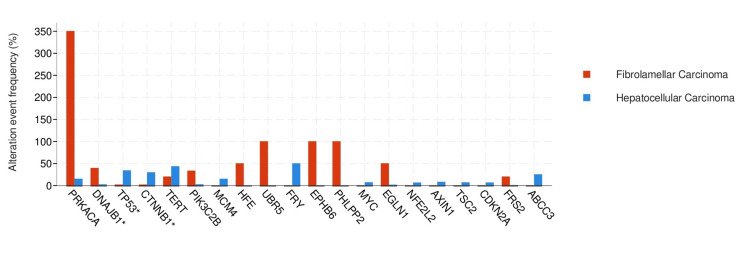
Pivotal genomic alterations (mutations, structural variants, and copy number alterations) Blue bars represent hepatocellular carcinoma and red bars represent fibrolamellar hepatocellular carcinoma. The data is conveyed in terms of percentages (%). Statistically significant variation existed between the groups (q<0.01).

The median (Q1-Q3) for the mutational count was 2 (1-4) in the FL-HCC group and 6 (3-9) in the HCC group (p<0.05). In terms of fractional genome alteration, the median (Q1-Q3) was 0.05 (0.01-0.17) in FL-HCC and 0.18 (0.06-0.31) in the HCC group. Genomic alteration was statistically significantly different in the FL-HCC and HCC groups (q<0.05)(Figure 2).

**Figure 3:**
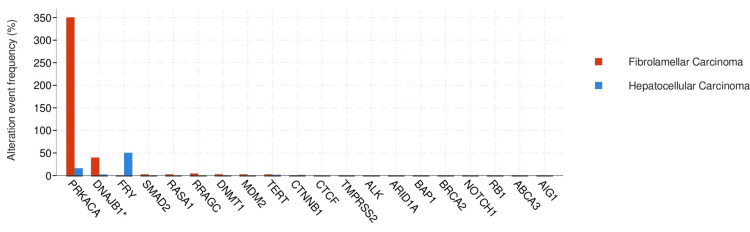
Structural variants/fusions with the most significant value Blue bars represent hepatocellular carcinoma and red bars represent fibrolamellar hepatocellular carcinoma. The data has been represented as percentages (%). Difference between the groups was statistically significant (q<0.01)*.*

In the HCC group, TP53 and CTNNB1 mutations were frequently observed. TP53 mutation was observed in 33.3% of the HCC group and 1.96 of the FL-HCC group. CTNNB1 was observed in 28.98% of the HCC group and 1.96% in FL-HCC. Variations in mutated genes between the two groups exhibited statistical significance (q<0.05). DNAJB1 fusion was observed in 40% of FL-HCC and 1.18% in the HCC group (q<0.05) (Figure 3 )

## Discussion

This study elucidated the genomic disparities distinguishing FL-HCC from conventional HCC, representing, to the best of our knowledge, extensive investigation encompassing FL-HCC patients conducted thus far. FL-HCC demonstrated a lower mutational count and fractional genome alteration compared to conventional HCC.

In this study, gender distribution also emerged as a distinctive factor, with a similar distribution between male and female patients in FL-HCC, contrary to the male predominance observed in conventional HCC. In the literature, it is reported that HCC was a male-predominant disease [14]. Eggert et al. reported male predominance in all subtypes of HCC but the lowest male-to-female ratio was 1.7 in FL-HCC [15].

Moreover, genomic analysis revealed distinct mutation patterns between FL-HCC and conventional HCC. Notably, while conventional HCC frequently displayed mutations in the *TP53 *and *CTNNB1 *genes, FL-HCC exhibited a higher prevalence of DNAJB1 fusion, further highlighting the unique genomic landscape specific to FL-HCC. These findings underscore the importance of comprehensive molecular characterization in distinguishing between HCC subtypes and elucidating the unique genetic underpinnings of FL-HCC.

Six principal biological pathways were shown in liver carcinogenesis. These pathways encompass telomere maintenance, Wnt/b-catenin signaling, TP53-mediated cell cycle regulation, oxidative stress response, epigenetic modification mechanisms, and AKT/mTOR and mitogen-activated protein (MAP) kinase pathways. The most frequent mutations and chromosome alterations were identified in the TERT promoter, CTNNB1, TP53, ARID1A, ARID2, NFE2L2, AXIN1, and RPS6KA3 in HCC [16]. In the literature, DNA sequencing and mutation analysis revealed a set of notably mutated genes encompassing *LZTR1*, *EEF1A1*, *SF3B1*, and *SMARCA4 *[17]. Nia et al. also reported that driver mutations in pathways like tumor suppressor genes, telomerase, the WNT-beta catenin pathway, PI3K/AKT/mTOR pathways, myelocytomatosis viral-related oncogene (MYC) pathway, JAK/STAT (Janus kinase/signal transducers and activators of transcription) pathways, oxidative stress pathways, Ras**/**rapidly accelerated fibrosarcom (RAF)​​​​​​​/mitogen-activated protein (MAP) kinases, and the Mesenchymal-to-epithelial transition (MET)​​​​​​​pathway [18]. Howell et al. also reported that the most frequent genes were *ARID1A*, *CTNNB1*, and *TP53 *in plasma cell-free circulating tumor DNA [19].

DNAJB1-PRKACA is specific to FL-HCC. Honeyman et al. initially identified the fusion gene DNAJB1-PRKACA through comprehensive analyses involving whole-transcriptome and whole-genome sequencing of paired tumor and adjacent normal liver samples [11]. Cornella et al. also reported that DNAJB1-PRKACA was detected in nearly 80% of the FL-HCC samples [20]. Given its potentially pivotal role in the development of FL-HCC, DNAJB1-PRKACA is a promising candidate for targeted therapy or as a prognostic marker.

There are a few limitations in this study. These limitations encompass the retrospective design of the study, the absence of exhaustive patient clinical and genomic attributes such as comorbidities, mRNA (messenger RNA) and miRNA (micro RNA) sequencing, and the deficiency in treatment and survival data. Future inquiries should delve deeper into elucidating the interplay between genomic attributes, treatment efficacy, and survival metrics across diverse HCC subtypes.

## Conclusions

In summary, this investigation underscores the considerable genomic disparities evident between FL-HCC and HCC across a substantial cohort of patients. The discernible clinical, histological, and genomic differences between these two variants of HCC underscore the significance of delineating and comprehending these genetic variations to expand the scope of available treatment modalities. The identification of these specific genomic alterations will lay the groundwork for developing more precise and effective therapeutic approaches. However, comprehensive studies are warranted to meticulously delineate specific genomic modifications and their intricate correlations with overall survival outcomes and treatment modalities.
